# Reply to Thet et al. Comment on “Oehler et al. Outcome and Midterm Survival after Heart Transplantation Is Independent from Donor Length of Stay in the Intensive Care Unit. *Life* 2022, *12*, 1053”

**DOI:** 10.3390/life13071444

**Published:** 2023-06-26

**Authors:** Daniel Oehler, Charlotte Böttger, Moritz Benjamin Immohr, Raphael Romano Bruno, Jafer Haschemi, Daniel Scheiber, Patrick Horn, Hug Aubin, Igor Tudorache, Ralf Westenfeld, Payam Akhyari, Malte Kelm, Artur Lichtenberg, Udo Boeken

**Affiliations:** 1 Division of Cardiology, Pulmonology and Vascular Medicine Medical Faculty, Heinrich-Heine University, 40225 Düsseldorf, Germany; 2 Department of Diagnostic and Interventional Radiology, Medical Faculty, Heinrich-Heine University, 40225 Düsseldorf, Germany; 3 Department of Cardiac Surgery, Medical Faculty, Heinrich-Heine University, 40225 Düsseldorf, Germany

Myat Soe Thet et al. published a letter [1] to the editor highlighting additional aspects that were not addressed in full in our article [2].

Thet et al. highlight that we only included donors with donation after brain death (DBD). This may certainly have impacted outcomes like length of stay of the donor prior to HTX. Due to legal constraints, transplant centers in Germany can only accept donors in the setting of DBD. In the whole Eurotransplant area, the absolute number of donors with donation after circulatory death (DCD) used for heart transplantations has been increasing over the recent years, from *n* = 4 in 2019 to *n* = 25 in 2022 [3]. However, those are still low in absolute numbers in comparison to all heart transplants in the same area (*n* = 629 in 2022) [4], and in the recently published Canadian DONATE study [5] by D’Aragon et al., no heart transplantation at all was performed from donors with DCD. Thus, the potential confounder bias of DCD vs. DBD on outcome after HTx in our reference cohorts seems to be negligible. However, we agree that we cannot fully exclude a potential bias here. This could be best ruled out by multicenter studies, potentially also across the current transplant systems worldwide, in the future, only.

Concerning the point of more commonly mild hypernatremia among the 35 donors with medium LOS in our study, Thet et al. are referring to the ongoing debate about the influence of donor sodium levels and our previous work in that field [6]. Therefore, we agree that hypernatremia could be a potential confounder here, which we cannot exclude totally.

Thet et al. also mentioned that we reported lower hemoglobin levels in the subgroup with the longest LOS in the ICU as compared with the other two subgroups (9 g/dL vs. 11 g/dL and 10 g/dL). We agree as we have discussed this interesting finding in our original work: it could be speculated that blood transfusion triggers were set at lower levels than usual around the time of brain death, which is supported by the data that show that, usually in critically ill patients, a longer LOS in the ICU is associated with a higher rate of transfusion of red blood cells due to decreasing hemoglobin levels [7]. As unfortunately we were limited by what information we could gather from the donor report, we cannot investigate further explanations, but we agree that this finding is worthwhile to be studied in future works in that field.

Regarding our findings and discussion that donors with the longest duration in the ICU had less frequent CPR prior to organ donation, this can be explained by the link between higher and earlier mortality in patients with CPR in the ICU [8], reducing the length of stay in this cohort. In other words, those receiving early brain death are then also earlier available for organ donation. Thet et al. also mentioned that there were some other parameters such as numerically differing rates of infection; however, none of them were significantly different. Nevertheless, through the retrospective single-center character, there is no absolute guarantee that one of those factors could be a potential confounder to be identified in future prospective studies.

Additionally, the rate of mechanical circulatory support and prior sternotomies is of interest. We thank Thet et al. for those questions and agree that those are important variables to compare between the groups. In the original publication, we already stated the rate of ventricular assist device, with no relevant difference between the three groups (50 vs. 50 vs. 52%). The rate of cardiac reoperation prior to HTx (including aortic and mitral valve replacement, coronary bypass surgery and LVAD as well as RVAD implantation), also stated in the original work, includes mainly sternotomies and did not show big differences between all three groups either (62 vs. 65 vs. 62%). Notably, the rate of Re-sternotomies post-HTx in our cohort was also not significantly different (29.7 vs. 28.8 vs. 31.8%, *p* > 0.72 in all comparisons). Additionally, the rate of ECLS in recipients prior to HTx, previously not reported, was also comparable between the groups (shortest to longest stay: 4.3 vs. 3.7 vs. 4.9%).

Primary graft dysfunction (PGD) is known to be associated with donor brain stem death and donor heart dysfunction. However, the rate of PGD in our cohort was previously not reported within the original publication. We analyzed the mortality rate in regard to that, and there was no relevant difference between the groups (from shortest to longest LOS): Group 0:4/27 (14.8%), Group 1:4/27 (14.8%), Group 2:3/23 (13.0%). Therefore, the brain-stem-PGD-axis seems not to play an important role here in explaining the difference between the three groups. However, to further elucidate the potential impact on survival, future studies in larger cohorts and best in a prospective and multicenter setting are needed. 

As Thet et al. mentioned, local donor and recipient factors may play a substantial role in predicting outcome in heart transplant recipients, while current scoring systems are unable to predict risk of donor–recipient pairs sufficiently. We agree on that point, and as we and others are working on integrating our findings, we hope that in future we will be able to perform center-wise risk stratification on the individual level better than it is possible currently.

We appreciate the recommendations of Myat Soe Thet et al. for further reading of our article by a broad readership. We are looking forward to RCTs that may confirm our observations, as donor selection is still one of the crucial topics in heart transplantation. 
